# Tetra­aqua­bis­[3-(pyridin-4-yl)benzoato-κ*N*]manganese(II)

**DOI:** 10.1107/S1600536812014602

**Published:** 2012-04-13

**Authors:** Ru-Qin Gao, Guo-Ting Li

**Affiliations:** a Department of Environmental and Municipal Engineering, North China University of Water Conservancy and Electric Power, Zhengzhou 450011, People’s Republic of China

## Abstract

In the title compound, [Mn(C_12_H_8_NO_2_)_2_(H_2_O)_4_], the Mn^2+^ ion lies on a twofold rotation axis and has a distorted N_2_O_4_ octa­hedral coordination geometry formed by four water O atoms in the equatorial plane and two apical pyridyl N atoms. A three-dimensional network is formed in the crystal structure by multiple O—H⋯O hydrogen bonds between the coordin­ating water molecules and the free carboxylate groups.

## Related literature

For pyrid­yl–multicarboxyl­ate–metal frameworks, see: Huang *et al.* (2007 ▶). For 3-pyridin-4-yl­benzo­ate compounds, see: Wu *et al.* (2011 ▶) For the isotypic Co complex, see: Wang & Li (2011 ▶).
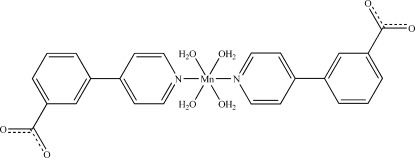


## Experimental

### 

#### Crystal data

[Mn(C_12_H_8_NO_2_)_2_(H_2_O)_4_]
*M* *_r_* = 523.39
Monoclinic, 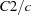

*a* = 24.935 (3) Å
*b* = 7.1911 (6) Å
*c* = 13.9283 (16) Å
β = 112.199 (13)°
*V* = 2312.4 (4) Å^3^
*Z* = 4
Mo *K*α radiation
μ = 0.63 mm^−1^
*T* = 293 K
0.24 × 0.20 × 0.16 mm

#### Data collection

Siemens SMART CCD diffractometer
Absorption correction: multi-scan (*SADABS*; Sheldrick, 1996 ▶) *T*_min_ = 0.875, *T*_max_ = 0.913
4456 measured reflections
2035 independent reflections
1673 reflections with *I* > 2σ(*I*)
*R*_int_ = 0.028

#### Refinement

*R*[*F* ^2^ > 2σ(*F* ^2^)] = 0.034
*wR*(*F* ^2^) = 0.079
*S* = 1.05
2035 reflections
171 parameters
H atoms treated by a mixture of independent and constrained refinement
Δρ_max_ = 0.19 e Å^−3^
Δρ_min_ = −0.20 e Å^−3^

### 

Data collection: *SMART* (Siemens, 1996 ▶); cell refinement: *SAINT* (Siemens, 1994 ▶); data reduction: *SAINT*; program(s) used to solve structure: *SHELXS97* (Sheldrick, 2008 ▶); program(s) used to refine structure: *SHELXL97* (Sheldrick, 2008 ▶); molecular graphics: *XP* in *SHELXTL* (Sheldrick, 2008 ▶); software used to prepare material for publication: *SHELXL97*.

## Supplementary Material

Crystal structure: contains datablock(s) I, global. DOI: 10.1107/S1600536812014602/bt5868sup1.cif

Structure factors: contains datablock(s) I. DOI: 10.1107/S1600536812014602/bt5868Isup2.hkl

Additional supplementary materials:  crystallographic information; 3D view; checkCIF report

## Figures and Tables

**Table 1:** Hydrogen-bond geometry (Å, °)

*D*—H⋯*A*	*D*—H	H⋯*A*	*D*⋯*A*	*D*—H⋯*A*
O3—H3*A*⋯O2^i^	0.86 (2)	1.91 (2)	2.732 (2)	160 (2)
O3—H3*B*⋯O1^ii^	0.80 (2)	1.92 (3)	2.715 (2)	175 (2)
O4—H4*A*⋯O1^iii^	0.86 (3)	1.86 (3)	2.728 (2)	176 (2)
O4—H4*B*⋯O2^iv^	0.84 (2)	1.92 (2)	2.726 (2)	161 (2)

Symmetry codes: (i) 
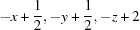
; (ii) 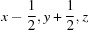; (iii) 
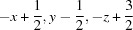
; (iv) 
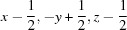
.

**Fig. 1.**
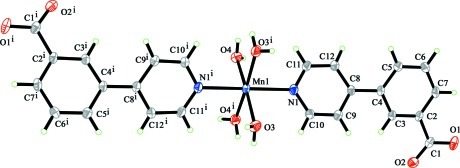
ORTEP diagram of (1) with atom numbering scheme (30% probability ellipsoids for all non-hydrogen atoms). Symmetry code: (i) -x, y, -z + 3/2.

**Fig. 2.**
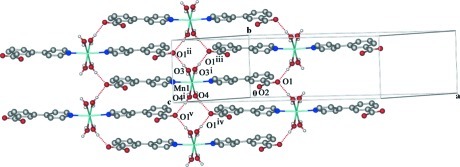
View of regular arrangement of monomers (1) directed by strong hydrogen bonding to form two-dimensional layers and face-to-face π···π stacking interactions between adjacent benzoicate and pyridyl groups of PBC. Symmetry codes: (i) -x, y, -z + 3/2; (ii) x - 1/2, y + 1/2, z; (iii) -x + 1/2, y + 1/2, -z + 3/2; (iv) -x + 1/2, y - 1/2, -z + 3/2; (v) x - 1/2, y - 1/2, z.

## supplementary crystallographic information

### Comment

Pyridyl-containing multi-carboxylic acids have been extensively investigated on the construction of various metal-organic frameworks (Huang *et al.*, 2007). Pyridylbenzoate ligands which possess a pyridyl group and a benzoic acid group are typical unsymmetrical spacers. Very recently, a serial of coordination polymers of 3-pyridin-4-ylbenzoic acid (PBC) was synthesized and characterized (Wu, *et al.*, 2011). Herein we report a new Mn(II) complex with (PBC), namely, [Mn(PBC)_2_(H_2_O)_4_] (1) which is isostructural with the complex [Co(PBC)_2_(H_2_O)_4_] (Wang & Li, 2011).

As showed in Fig. 1, (1) is a mononuclear complex with a twofold axis passing through the Mn(II) center along *b* axis and equally splitting the whole molecule. In (1) the Mn(II) center is ligated by four O of coordinated water molecules in the equatorial plane, and two PBC acting as monodentate ligands occupy the axial positions through their pyridyl nitrogen atoms coordinating to Mn(II). Thus the Mn(II) ion is in a six-coordinated octahedral geometry. The bond distances of Mn—O and Mn—N range from 2.1867 (16) to 2.2661 (16) Å, while the in-plane and axis-transition angles are 173.04 (6) and 175.06 (8) °, respectively, indicating a slight distortion of the octahedral coordination sphere around the Mn(II) center.

Further aggregation of the monomers (1) is formed by the multiple hydrogen-bonding between the coordinated water molecules (as donors) and the uncoordinated carboxylate groups (as acceptors) (Table 1). Hydrogen-bonding system among monomers (1) is rather complicated: each water molecule forms two O—H···O hydrogen bonds with carboxylate groups of neighbouring complex molecules, while every carboxylate group of PBC forms three hydrogen bonds. Consequently, every monomer acts as a novel six-connected supramolecular synthon to connect with six adjacent monomers. Notably, the hydrogen-bonding models of the carboxyl group of PBC play an important role in the formation of crystal structure of (1). For example, as shown in Fig. 2, the O1 atom of the carboxylate group of PBC in a hydrogen-bonding bridging mode ligates to two water molecules from two neighboring monomers, and as a result, monomers (1) are regularly arrayed in *ab* plane and linked into two-dimensional layers by strong hydrogen bonding (O3···O1, 2.715 (2) Å; O4···O1, 2.728 (2) Å). The layer structure is stabilized by forceful face-to-face π···π stacking interactions between adjacent benzoicate groups and pyridyl groups of PBC with a centroid to centroid distance of 3.62 (1) Å. Intriguingly, the benzoicate group and pyridyl group of PBC distort to 27.6 (0) ° to meet the formation of hydrogen bonding. The layers are further bound together to create the three-dimensional supramolecular architecture by hydrogen bonds between the O2 atom of the carboxylate group of PBC and two water molecules in the adjacent complex molecue. monomer.

### Experimental

The title compound, (1), was prepared according to the following process. A mixture of MnCO_3_ (0.012 g, 0.1 mmol), PBC (0.040 g, 0.2 mmol) and deionized water (10 ml) was sealed into a 25 ml Teflon-lined stainless autoclave. The autoclave was heated at 160 °C for four days. As cooled to room temperature gradually, pale yellow needle crystals of (1) suitable for X-ray analysis were obtained in 64% yield (based on Mn).

### Refinement

All H atoms were located in a difference map. The coordinates of the water H atoms were refined with U(H) set to 1.2U_eq_(O). H atoms bonded to C were refined as riding with C-H = 0.95Å and 1.2U_eq_(C).

### Crystal data

**Table:**

[Mn(C_12_H_8_NO_2_)_2_(H_2_O)_4_]	*F*(000) = 1084
*M**_r_* = 523.39	*D*_x_ = 1.503 Mg m^−^^3^
Monoclinic, *C*2/*c*	Mo *K*α radiation, λ = 0.71073 Å
Hall symbol: -C 2yc	Cell parameters from 1769 reflections
*a* = 24.935 (3) Å	θ = 3.0–27.6°
*b* = 7.1911 (6) Å	µ = 0.63 mm^−^^1^
*c* = 13.9283 (16) Å	*T* = 293 K
β = 112.199 (13)°	Needle, yellow
*V* = 2312.4 (4) Å^3^	0.24 × 0.20 × 0.16 mm
*Z* = 4	

### Data collection

**Table:**

Siemens SMART CCD diffractometer	2035 independent reflections
Radiation source: fine-focus sealed tube	1673 reflections with *I* > 2σ(*I*)
Graphite monochromator	*R*_int_ = 0.028
ω scan	θ_max_ = 25.0°, θ_min_ = 3.0°
Absorption correction: multi-scan (*SADABS*; Sheldrick, 1996)	*h* = −29→29
*T*_min_ = 0.875, *T*_max_ = 0.913	*k* = −5→8
4456 measured reflections	*l* = −16→16

### Refinement

**Table:**

Refinement on *F*^2^	Primary atom site location: structure-invariant direct methods
Least-squares matrix: full	Secondary atom site location: difference Fourier map
*R*[*F*^2^ > 2σ(*F*^2^)] = 0.034	Hydrogen site location: inferred from neighbouring sites
*wR*(*F*^2^) = 0.079	H atoms treated by a mixture of independent and constrained refinement
*S* = 1.05	*w* = 1/[σ^2^(*F*_o_^2^) + (0.0389*P*)^2^] where *P* = (*F*_o_^2^ + 2*F*_c_^2^)/3
2035 reflections	(Δ/σ)_max_ < 0.001
171 parameters	Δρ_max_ = 0.19 e Å^−^^3^
0 restraints	Δρ_min_ = −0.20 e Å^−^^3^

### Special details

**Table:**

Geometry. All e.s.d.'s (except the e.s.d. in the dihedral angle between two l.s. planes) are estimated using the full covariance matrix. The cell e.s.d.'s are taken into account individually in the estimation of e.s.d.'s in distances, angles and torsion angles; correlations between e.s.d.'s in cell parameters are only used when they are defined by crystal symmetry. An approximate (isotropic) treatment of cell e.s.d.'s is used for estimating e.s.d.'s involving l.s. planes.
Refinement. Refinement of *F*^2^ against ALL reflections. The weighted *R*-factor *wR* and goodness of fit *S* are based on *F*^2^, conventional *R*-factors *R* are based on *F*, with *F* set to zero for negative *F*^2^. The threshold expression of *F*^2^ > σ(*F*^2^) is used only for calculating *R*-factors(gt) *etc*. and is not relevant to the choice of reflections for refinement. *R*-factors based on *F*^2^ are statistically about twice as large as those based on *F*, and *R*- factors based on ALL data will be even larger.

### Fractional atomic coordinates and isotropic or equivalent isotropic displacement parameters (Å^2^)

**Table:**

	*x*	*y*	*z*	*U*_iso_*/*U*_eq_	
Mn1	0.0000	0.30640 (6)	0.7500	0.03055 (16)	
O1	0.46120 (6)	0.2912 (2)	0.87784 (13)	0.0494 (4)	
O2	0.40494 (7)	0.2263 (2)	0.96408 (12)	0.0557 (5)	
O3	0.03234 (7)	0.5054 (2)	0.87792 (12)	0.0434 (4)	
O4	−0.02832 (7)	0.0811 (2)	0.63641 (12)	0.0414 (4)	
N1	0.08789 (7)	0.3200 (2)	0.73735 (13)	0.0343 (4)	
C1	0.41251 (9)	0.2746 (3)	0.88379 (18)	0.0373 (5)	
C2	0.35888 (8)	0.3161 (3)	0.78874 (16)	0.0307 (5)	
C3	0.30485 (8)	0.3071 (3)	0.79533 (16)	0.0304 (4)	
H3	0.3024	0.2747	0.8597	0.036*	
C4	0.25382 (8)	0.3442 (3)	0.71007 (15)	0.0296 (5)	
C5	0.25909 (9)	0.3921 (3)	0.61674 (16)	0.0372 (5)	
H5	0.2253	0.4182	0.5574	0.045*	
C6	0.31246 (9)	0.4019 (3)	0.60960 (16)	0.0422 (6)	
H6	0.3151	0.4351	0.5455	0.051*	
C7	0.36272 (9)	0.3639 (3)	0.69523 (17)	0.0372 (5)	
H7	0.3995	0.3707	0.6895	0.045*	
C8	0.19674 (8)	0.3344 (3)	0.71925 (15)	0.0294 (5)	
C9	0.19034 (9)	0.3677 (3)	0.81302 (16)	0.0368 (5)	
H9	0.2233	0.3967	0.8732	0.044*	
C10	0.13683 (9)	0.3587 (3)	0.81876 (17)	0.0384 (5)	
H10	0.1341	0.3812	0.8840	0.046*	
C11	0.09364 (9)	0.2880 (3)	0.64725 (17)	0.0362 (5)	
H11	0.0598	0.2597	0.5884	0.043*	
C12	0.14589 (9)	0.2939 (3)	0.63492 (16)	0.0364 (5)	
H12	0.1473	0.2703	0.5688	0.044*	
H3A	0.0499 (10)	0.451 (3)	0.9365 (18)	0.055*	
H4A	−0.0072 (10)	−0.013 (3)	0.6343 (18)	0.055*	
H3B	0.0120 (11)	0.588 (3)	0.8818 (18)	0.055*	
H4B	−0.0448 (10)	0.126 (3)	0.5770 (19)	0.055*	

### Atomic displacement parameters (Å^2^)

**Table:**

	*U*^11^	*U*^22^	*U*^33^	*U*^12^	*U*^13^	*U*^23^
Mn1	0.0213 (3)	0.0359 (3)	0.0354 (3)	0.000	0.0119 (2)	0.000
O1	0.0229 (8)	0.0438 (9)	0.0791 (12)	−0.0003 (7)	0.0165 (8)	0.0015 (8)
O2	0.0358 (9)	0.0824 (12)	0.0427 (9)	−0.0011 (8)	0.0076 (8)	0.0058 (9)
O3	0.0351 (10)	0.0453 (10)	0.0458 (10)	0.0083 (7)	0.0107 (8)	−0.0068 (8)
O4	0.0409 (10)	0.0388 (9)	0.0435 (9)	0.0052 (7)	0.0147 (8)	−0.0010 (7)
N1	0.0263 (9)	0.0370 (10)	0.0409 (10)	0.0025 (8)	0.0139 (8)	0.0045 (8)
C1	0.0272 (12)	0.0313 (11)	0.0493 (13)	−0.0015 (10)	0.0098 (10)	−0.0065 (10)
C2	0.0238 (11)	0.0273 (10)	0.0420 (12)	−0.0025 (9)	0.0136 (9)	−0.0063 (9)
C3	0.0271 (11)	0.0300 (10)	0.0365 (11)	−0.0015 (9)	0.0149 (9)	−0.0028 (9)
C4	0.0254 (11)	0.0277 (11)	0.0388 (11)	−0.0013 (9)	0.0157 (9)	−0.0013 (9)
C5	0.0293 (12)	0.0440 (12)	0.0368 (12)	−0.0019 (10)	0.0110 (10)	0.0020 (10)
C6	0.0404 (14)	0.0547 (14)	0.0383 (12)	−0.0023 (12)	0.0227 (11)	0.0028 (11)
C7	0.0294 (12)	0.0384 (12)	0.0508 (13)	−0.0050 (10)	0.0229 (11)	−0.0056 (10)
C8	0.0248 (11)	0.0276 (11)	0.0373 (11)	0.0021 (9)	0.0134 (9)	0.0050 (9)
C9	0.0255 (11)	0.0453 (12)	0.0387 (12)	−0.0006 (10)	0.0110 (10)	−0.0005 (10)
C10	0.0287 (12)	0.0524 (13)	0.0366 (11)	0.0023 (10)	0.0152 (10)	0.0000 (10)
C11	0.0241 (11)	0.0423 (12)	0.0398 (12)	0.0009 (10)	0.0094 (9)	0.0006 (10)
C12	0.0295 (12)	0.0443 (12)	0.0377 (11)	0.0001 (10)	0.0154 (10)	−0.0011 (10)

### Geometric parameters (Å, º)

**Table:**

Mn1—O4	2.1867 (16)	C3—C4	1.400 (3)
Mn1—O4^i^	2.1867 (16)	C3—H3	0.9500
Mn1—O3	2.1878 (16)	C4—C5	1.398 (3)
Mn1—O3^i^	2.1878 (16)	C4—C8	1.478 (3)
Mn1—N1	2.2661 (16)	C5—C6	1.373 (3)
Mn1—N1^i^	2.2661 (16)	C5—H5	0.9500
O1—C1	1.253 (2)	C6—C7	1.392 (3)
O2—C1	1.252 (3)	C6—H6	0.9500
O3—H3A	0.86 (2)	C7—H7	0.9500
O3—H3B	0.80 (2)	C8—C9	1.395 (3)
O4—H4A	0.86 (3)	C8—C12	1.395 (3)
O4—H4B	0.84 (2)	C9—C10	1.368 (3)
N1—C11	1.336 (3)	C9—H9	0.9500
N1—C10	1.344 (3)	C10—H10	0.9500
C1—C2	1.514 (3)	C11—C12	1.378 (3)
C2—C7	1.385 (3)	C11—H11	0.9500
C2—C3	1.386 (3)	C12—H12	0.9500
			
O4—Mn1—O4^i^	84.40 (9)	C2—C3—C4	121.98 (18)
O4—Mn1—O3	173.04 (6)	C2—C3—H3	119.0
O4^i^—Mn1—O3	88.64 (6)	C4—C3—H3	119.0
O4—Mn1—O3^i^	88.64 (6)	C5—C4—C3	117.47 (18)
O4^i^—Mn1—O3^i^	173.04 (6)	C5—C4—C8	121.60 (18)
O3—Mn1—O3^i^	98.31 (10)	C3—C4—C8	120.92 (18)
O4—Mn1—N1	91.89 (6)	C6—C5—C4	120.93 (19)
O4^i^—Mn1—N1	91.76 (6)	C6—C5—H5	119.5
O3—Mn1—N1	88.02 (6)	C4—C5—H5	119.5
O3^i^—Mn1—N1	88.75 (6)	C5—C6—C7	120.74 (19)
O4—Mn1—N1^i^	91.76 (6)	C5—C6—H6	119.6
O4^i^—Mn1—N1^i^	91.89 (6)	C7—C6—H6	119.6
O3—Mn1—N1^i^	88.75 (6)	C2—C7—C6	119.66 (19)
O3^i^—Mn1—N1^i^	88.02 (6)	C2—C7—H7	120.2
N1—Mn1—N1^i^	175.06 (8)	C6—C7—H7	120.2
Mn1—O3—H3A	112.0 (16)	C9—C8—C12	115.78 (18)
Mn1—O3—H3B	119.7 (18)	C9—C8—C4	121.83 (18)
H3A—O3—H3B	113 (2)	C12—C8—C4	122.39 (18)
Mn1—O4—H4A	124.7 (16)	C10—C9—C8	120.39 (19)
Mn1—O4—H4B	109.7 (17)	C10—C9—H9	119.8
H4A—O4—H4B	110 (2)	C8—C9—H9	119.8
C11—N1—C10	116.28 (18)	N1—C10—C9	123.71 (19)
C11—N1—Mn1	121.06 (14)	N1—C10—H10	118.1
C10—N1—Mn1	122.66 (14)	C9—C10—H10	118.1
O2—C1—O1	124.2 (2)	N1—C11—C12	123.65 (19)
O2—C1—C2	117.03 (19)	N1—C11—H11	118.2
O1—C1—C2	118.8 (2)	C12—C11—H11	118.2
C7—C2—C3	119.22 (19)	C11—C12—C8	120.20 (19)
C7—C2—C1	121.28 (19)	C11—C12—H12	119.9
C3—C2—C1	119.50 (18)	C8—C12—H12	119.9

Symmetry code: (i) −*x*, *y*, −*z*+3/2.

### Hydrogen-bond geometry (Å, º)

**Table:**

*D*—H···*A*	*D*—H	H···*A*	*D*···*A*	*D*—H···*A*
O3—H3*A*···O2^ii^	0.86 (2)	1.91 (2)	2.732 (2)	160 (2)
O3—H3*B*···O1^iii^	0.80 (2)	1.92 (3)	2.715 (2)	175 (2)
O4—H4*A*···O1^iv^	0.86 (3)	1.86 (3)	2.728 (2)	176 (2)
O4—H4*B*···O2^v^	0.84 (2)	1.92 (2)	2.726 (2)	161 (2)

Symmetry codes: (ii) −*x*+1/2, −*y*+1/2, −*z*+2; (iii) *x*−1/2, *y*+1/2, *z*; (iv) −*x*+1/2, *y*−1/2, −*z*+3/2; (v) *x*−1/2, −*y*+1/2, *z*−1/2.
